# Does Etiology Matter? Comparative Analysis of a Singing-Enhanced Swallowing Protocol for Patients with Neurological Impairment versus Head and Neck Cancer

**DOI:** 10.3390/brainsci11080997

**Published:** 2021-07-28

**Authors:** Myung Sun Yeo, Ga Eul Yoo, Sung-Rae Cho, Soo Ji Kim

**Affiliations:** 1 Department of Music Therapy, Graduate School, Ewha Womans University, Ewhayeodae-gil 52, Seodaemun-gu, Seoul 03760, Korea; odrose@naver.com (M.S.Y.); bbird27@ewha.ac.kr (G.E.Y.); 2 Ewha Music Rehabilitation Center, Ewha Womans University, Ewhayeodae-gil 52, Seodaemun-gu, Seoul 03760, Korea; 3 Department and Research Institute of Rehabilitation Medicine, Yonsei University College of Medicine, Yosei-ro 50-1, Seodaemun-gu, Seoul 03722, Korea; srcho918@yuhs.ac; 4 Brain Korea 21 PLUS Project for Medical Science, Yonsei University College of Medicine, Seoul 03722, Korea; 5 Music Therapy Education, Graduate School of Education, Ewha Womans University, Ewhayeodae-gil 52, Seodaemun-gu, Seoul 03760, Korea

**Keywords:** dysphagia, singing, laryngeal elevation, head and neck cancer, Parkinson’s disease

## Abstract

Swallowing difficulties are a common complaint among patients with a variety of diseases. To address these concerns, a singing-enhanced swallowing protocol was constructed, and its differential benefits for two patient populations were investigated. Two patients with Parkinson’s disease (PD) and two patients with head and neck cancer (HNC) participated in this study. Each patient participated in 30-min individual sessions of a singing-enhanced swallowing protocol two times per week for 12 weeks. Following the intervention, laryngeal diadochokinesis and quality-of-life measurements were found to be higher in all four patients. However, the Videofluoroscopic Dysphagia Scale showed this improvement was associated with different swallowing tasks for each patient group. In addition, the maximum phonation time decreased for patients with HNC, while it increased for patients with PD. The findings support the use of a singing-enhanced swallowing protocol for patients whose swallowing difficulties are due to neurological or structural impairment. In addition, the study results suggest that different intervention components should be considered depending on the etiology of the patient’s swallowing difficulties.

## 1. Introduction

Difficulty in swallowing is a severe and even life-threatening condition. While there are various causes of dysphagia [1], it commonly leads to malnutrition and the degradation of a patient’s immune functions. It is associated with dehydration, laryngeal penetration, and aspiration pneumonia, which can delay patients’ functional recovery and limit their ability to independently engage in daily life activities [2]. Since dysphagia is a frequent complaint among patients with neurological impairment, various approaches to rehabilitative treatment for this population have been developed [3,4]. Although such attempts are advancing, their outcomes remain mixed [5,6], and efforts to examine these interventions with specific populations of patients with neurological disorders, such as patients with Parkinson’s disease (PD) [7], are only just beginning. Alongside patients with neurological impairment, patients with structural impairment (e.g., resulting from head and neck cancer [HNC]) are the most vulnerable to swallowing difficulties and resulting malnutrition [8]. Research on patients with HNC found that their swallowing difficulties were typically severe and even remained after the termination of their cancer treatment [9]. As a result, there is increasing interest in developing a rehabilitative intervention for this patient population. Unlike other diseases, the etiology of HNC, its treatment, and the complications associated with HNC treatment can all directly affect the patient’s swallowing mechanism, resulting in the need for a population-specific intervention.

Singing-based interventions have been introduced to address dysphagia-related symptoms and consequences [10,11,12]. Since singing requires breathing, vocalization, and articulation with a high respiration volume and vocalization intensity [13] and also involves the same organs that are used during speaking and swallowing, singing-based exercises have the potential to improve swallowing-related functions. In a preliminary study [14], a therapeutic singing technique was used to promote laryngeal elevation, which leads to the closure of the epiglottis and prevents aspiration during swallowing. The singing protocol was shown to enhance the swallowing function by strengthening the larynx muscles of patients with dysphagia.

Such laryngeal elevation-based singing techniques have recently been found to offer desirable outcomes for some patients with neurological impairment [15]. This is likely due to laryngeal movements that are involved in breathing and phonation as well as swallowing. Disorders that result in neurological impairment of the airway can disrupt swallowing apnea, which is one of the critical mechanisms that prevents airway aspiration [16]. For patients experiencing dysphagia due to neurological impairment, laryngeal elevation exercises that strengthen the larynx muscles are recommended alongside breathing or vocalization training. Such exercises can be adjusted to alter the intensity of the training and the difficulties of the task based on the specific needs of the patient with dysphagia and the etiology of the patient’s symptoms [14,17].

According to this perspective, singing can address the diverse needs of patients with dysphagia. Singing involves breathing and vocalization, and it can require a broad range of vocalization and articulatory movement depending on the type of syllables, words, or combination of words included in the lyrics or musical elements. Vocalization required for singing can improve the muscular functions of the articulation organs, such as the chin, the lips, and the tongue. In addition, the breathing control required for singing can strengthen the mobility and breathing functions of the facial and oral cavity muscles by inducing the coordination of vocalization organs as well as inducing regular and stable breathing [18]. Research has shown that repetition of vocalization with different musical notes assists maintenance of the elevation of the larynx through the up and down movement of the larynx [14]. With the possibility to adjust the multifaceted features of singing at different levels, singing for rehabilitation may differentially affect patients with different diagnoses (e.g., neurological or structural impairment).

Despite differences in etiology, the swallowing difficulties of patients with neurological impairment can resemble the swallowing difficulties of patients with structural impairment. It remains unclear whether singing-based interventions differentially impact patients based on the etiology of their dysphagia. As such, this study implemented a singing-enhanced swallowing protocol with two patient groups: patients whose dysphagia was due to neurological impairment (PD) and patients whose dysphagia was due to structural impairment (HNC) and compared the results of a singing-enhanced swallowing protocol for patients with different diagnoses in terms of outcomes and protocol considerations. The results of this study present baseline data and clinical implications for applying more diversified singing-based swallowing interventions for patients with dysphagia.

The research questions were the following:

Are there changes in the patients’ swallowing function, speech-language function, and swallowing-quality of life after their engagement in the singing-enhanced swallowing protocol?

Are there differences between the two patient groups (i.e., PD and HNC) in terms of their repetition rate for each component of the protocol?

## 2. Materials and Methods

### 2.1. Research Design

This research involved an empirical case study. Four participants were recruited. Before and after implementation of the protocol, observation-based measurements and task-based objective data were collected from the sample and analyzed in order to investigate changes in the patients following their participation in the protocol.

### 2.2. Participants

All procedures in this study were reviewed and approved by the Institutional Review Board of Severance Hospital (IRB No. 4-2012-0483). Four patients with dysphagia participated in this study: two were diagnosed with PD and two were diagnosed with HNC (one with tongue cancer and the other with mandibular gland cancer). The patients’ mean age was 65.8 years (ranging from 54 to 77 years), and the duration since diagnosis ranged from 3 to 7 years. Prior to their participation, each patient completed the informed consent form. Patients’ demographic information is displayed in Table 1.

### 2.3. Procedures

Each patient participated in individual 30-min sessions of the singing-enhanced swallowing protocol two times per week for 12 weeks. Some sessions were missed due to patient illness, resulting in patients receiving 20 to 24 sessions. Pretest measures were administered during the week prior to the patient’s first intervention session, and posttest measures were taken within 1 week after the patient completed the protocol.

### 2.4. Singing-Enhanced Swallowing Protocol

The singing-enhanced swallowing protocol consisted of five parts: respiratory muscle relaxation, vocal folds relaxation, laryngeal elevation, and two types of modified singing (i.e., singing focused on oral motor movement and respiration). The protocol was a modified version of the music-based swallowing enhancement protocol developed by one of the investigators of this study [12]. For the original protocol, swallowing exercises focused on laryngeal elevation as the primary activity. To investigate whether the swallowing enhancement protocol could effectively be applied to an expanded population, this study constructed a modified version of the protocol by adding singing that targeted oral motor movement and respiratory movement [15,19].

The first part of the protocol involved respiratory muscle relaxation. This was achieved through upper body movements: turning the neck right and left, lifting and lowering the shoulders, and stretching by fully extending the arms forward. These movements were accompanied by live keyboard playing, and the tempo and dynamics of the music were adjusted to match the movement executed by the patients and to provide a cue for each target movement.

The second part involved vocal folds relaxation and consisted of vocalizing single vowels, humming, and gliding a sound. This required the patients to vocalize the vowel /a/ while exhaling after inhaling sufficiently and holding their breath for a short while to achieve the effects of strengthening diaphragm resistance and lifting the soft palate. Since swallowing occurs during a state of momentary apnea, repetition of such training can help prevent airway aspiration. The patients were then instructed to hum or vocalize with a low voice to open-up the vocalization organs. Since humming can relax a tensed larynx and cause the vocal folds to become gradually introverted within a narrow range, it is appropriate as a preparatory process for vocalization [20].

Then, the patients executed vocal sound gliding using a single vowel from the lowest to the highest musical notes within their range. This aimed to induce the up and down movement of laryngeal muscles along with the movement of the oral cavity muscles; this can be deemed an appropriate activity for indirect observation of a patient since the range of the musical notes changes following the adjustment of the length of the vocal cords.

The third part of this protocol targeted laryngeal elevation, and this was the primary component of the protocol. This involved inducing laryngeal elevation by executing sequential vocalization of two vowels, /ooh/ and /i:/, following two different pitches. Each patient was instructed to vocalize the back vowel /ooh/ at a low pitch, the front vowel /i:/ at a high pitch, and both sounds with different musical pitches. Vocalizing from /ooh/ to /i:/ in a sequence without a break in phonation was repeated, which involves the movement of lingual muscles and larynx elevation as the tongue changes for each articulation position. Furthermore, jumping between two different pitches induces the elevation of laryngeal muscles [21]. This activity was designed to augment the upper esophageal sphincter (UES) opening by prolonging laryngeal elevation during swallowing. Maintaining this shape of the mouth may help to increase the duration of laryngeal elevation. While each patient was instructed to vocalize the two vowels differently by changing the pitch, they were assured that they did not have to produce an accurate pitch that matched the accompanying music (i.e., the piano).

Finally, the last two parts are the modified singing for respiratory control and for articulation based on the use of extended musical phrases or accents on regular beats in a song. For these parts, musical elements were modified to require controlled breathing or vocalization at different levels. While the patients’ preferred pieces were used, the original song’s lyrics, melody, or rhythm was modified. For respiratory control, repeated short phrases of melodies were included in the song while requiring regular breathing between phrases. For oral motor movement for articulation, repetition of a target vowel or a combination of syllables requiring patients to perform the target movement was included in the song.

### 2.5. Measures

#### 2.5.1. Videofluoroscopic Dysphagia Scale for Assessment of Swallowing Function

To analyze the changes in each patient’s swallowing function from pretest to posttest, a Videofluoroscopy Swallowing Study (VFSS) was administered, which recorded the patients engaging in swallowing tasks. Each patient swallowed three types of barium with the level of viscosity adjusted: 35% *w*/*v* of liquid barium solution with three-fold volume and two solid types of powder barium mixed at 12% (i.e., thick) and 6% (i.e., thin). A total of six swallowing tests were administered: 5 cc and 15 cc of high viscosity barium (thick solid), 5 cc and 15 cc of low viscosity barium (thin solid), and 5 cc and 15 cc of liquid barium (liquid). Each swallowing test was recorded at two phases (i.e., oral cavity phase and pharyngeal phase) and analyzed using the Videofluoroscopic Dysphagia Scale (VDS) [21]. Two evaluators (i.e., the first author and a researcher in rehabilitation medicine blind to the patients’ diagnoses) watched the recorded video and rated each patients’ swallowing function using the VDS. This scale consists of 14 items with seven items representing oral functions (i.e., closure of the lips, formation of food, mastication, swallowing apraxia, tongue-palate contact, early phase loss of food, and time for the swallowed substance to pass the oral cavity) and seven items representing pharyngeal functions (induction of pharynx swallowing, residue in the laryngeal flexure, laryngeal lift, residue in the pyriform sinus, pharyngeal wall coating, time for the swallowed substance to pass through the pharynx, and aspiration). The maximum score is 100, and a higher score signifies greater severity of dysphagia. The interrater reliability exceeded 90%.

#### 2.5.2. Voice Data Collection for Assessment of Speech-Language Function

Patients’ voice data were recorded in a quiet room with an ambient noise of less than 50 dB. A 20 cm distance was maintained between the mouth and the voice recorder, and the recorded voice data were analyzed using the Praat program. As a voice measure, maximum phonation time (MPT) was recorded. Each patient was asked to make the /ah/ sound and maintain it for as long as possible. Three trials of phonation were administered, and each patient’s longest MPT was included in the analysis. Furthermore, to evaluate the range and speed of movement of the vocal folds, each patient was asked to repeat the glottal syllable /∧/ as quickly, consistently, and accurately as possible for 5 s, and the rate of laryngeal diadochokinesis (L-DDK) was measured by calculating the number of syllables spoken per second [22,23,24,25].

Lastly, to evaluate patients’ oral motor articulatory function, alternating motion rate (AMR) and sequential motion rate (SMR) were measured. For the AMR measure, patients were asked to repeat /puh/, /tuh/, and /kuh/ as fast and as accurately as possible for 5 s. For SMR, the patients were asked to repeat /puh-tuh-kuh/ in a sequence over 5 s. The number of repeated target sounds during the 5 s was calculated.

#### 2.5.3. Swallowing Quality-of-Life Questionnaire

In this study, the Korean version of the Swallowing Quality-of-Life (SWAL-QOL) measure was used. The original SWAL-QOL has been validated in Korea and found to be a clinically valid and reliable tool for assessing the quality of life of Koreans with dysphagia [26]. SWAL-QOL is a 44-item tool that takes about 15 min to complete and assesses 10 quality-of-life concepts. It consists of eight dysphagia-related categories (food selection, burden, mental health, social functioning, fear, eating duration, eating desire, and communication) and two general quality-of-life categories (sleep and fatigue). The questions were intended to reflect the patient’s swallowing experience over the preceding month. SWAL-QOL also includes a 14-item symptom frequency battery, three questions related to nutritional intake, and one question on general health. The questions are scored on a 5-point Likert scale that can be transformed to scores ranging from 0 to 100 (least favorable to most favorable).

#### 2.5.4. Repetition Rate of Each Component in the Protocol

Each patient engaged in each of the five parts of the protocol. After completing the target tasks as planned, additional repetition of target tasks was requested if the patient did not complete the task to an expected level. While a lower repetition rate of a particular component indicates the patient’s higher performance in that part of the protocol, a higher repetition rate indicates lower performance with greater demand for that specific component.

## 3. Results

### 3.1. Data on the Swallowing Function

The results of the VDS are presented in Table 2. All four patients showed decreases in their VDS scores, particularly for the thick solid form of barium (both at 5 cc and 15 cc), which indicates an improved swallowing function. They also tended to swallow the thin solid form of barium more quickly at posttest, except one patient with PD (B) who showed no changes in their swallowing score for 15 cc of thin barium at any of the phases. For the liquid barium, there were group differences. Patients with PD (A and B) showed no changes for both 5 cc and 15 cc of liquid, while patients with HNC (C and D) showed decreases (i.e., improved swallowing function) when passing the 5 cc of liquid through the oral cavity.

### 3.2. Data on the Vocal and Speech-Language Functions

The results of vocal and speech-language function measures before and after the intervention are presented in Table 3.

**Figure 1:**
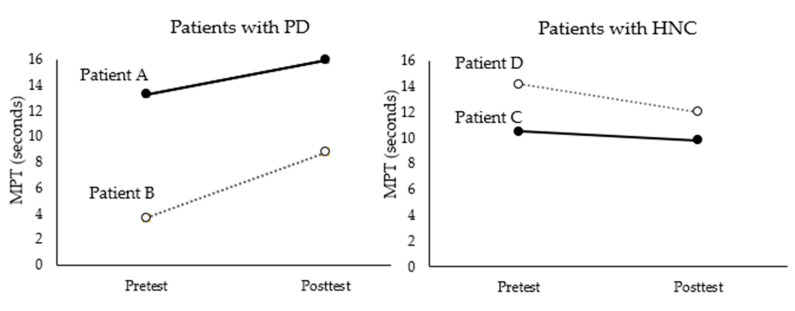
Results of MPT before and after intervention for each patient.

In terms of MPT, both patients A and B, who were diagnosed with PD, showed increases, while patients C and D, who were diagnosed with HNC, showed decreases (see Table 3 and Figure 1).

For AMR and SMR, which indicate the oral motor movement rate, patients generally showed increases in AMR, except for patient B who exhibited no change on the AMR subtask with /tuh/, and in each patient group, one patient (A and D) showed an increase on SMR for /puh-tuh-kuh/ while the other patients (B and C) showed no changes.

**Figure 2:**
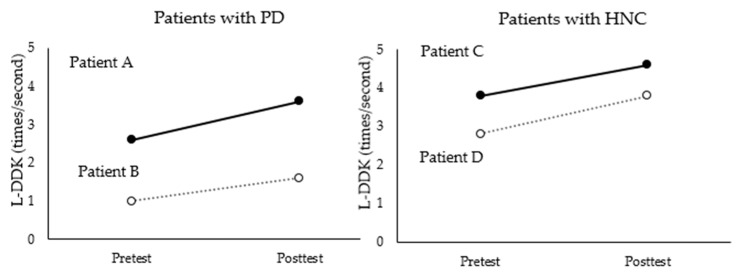
Results of L-DDK measurement before and after the intervention for each patient.

In terms of L-DDK, as illustrated in Figure 2, this measure increased after the intervention for all four patients. When it was converted into speed per second, greater increases were observed for patients with HNC (increase from 3.8 to 4.6 for patient D and from 2.8 to 3.8 for patient C) than for patients with PD (increase from 2.6 to 3.6 for patient A and from 1 to 1.6 for patient B).

### 3.3. Data on the SWAL-QOL

The SWAL-QOL scores are presented in Table 4. All of the patients’ total scores increased after the intervention. Items in which all patients showed improvement included burden of swallowing, sustained period of eating, and frequency of swallowing difficulties, which indicate that all patients perceived positive changes directly related to their swallowing-related symptoms.

### 3.4. Repetition Rate of Each Component within the Protocol

**Figure 3:**
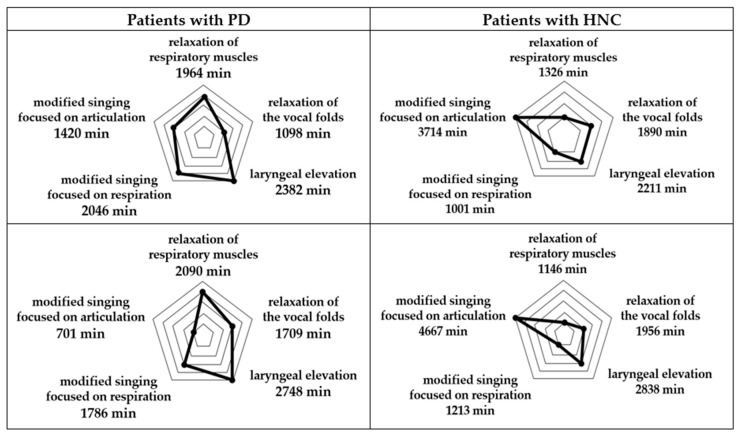
Repetition rate of each component within the protocol for each patient.

Additionally, we also analyzed the repetition rate of each component within the protocol when applying the task in order for the patient to complete the task at the expected level. Each target task related to the protocol component was categorized into relaxation of respiratory muscles, relaxation of the vocal folds, laryngeal elevation, or modified singing. An increased repetition rate indicates that the patient needed more time to complete the task at an expected level, which means the patient needed to practice that component more. Patients showed a clear difference in their repetition rates. It is interesting to note that patients A and B (with PD) demonstrated similar results as did patients C and D (with HNC), indicating that the diagnostic group may be a factor in these results (see Figure 3). While patients with PD spent more time on relaxation of respiratory muscles, singing focused on respiration, and laryngeal elevation, patients with HNC spent most of their time on oral motor movement-based singing. In other words, patients A and B (with PD) needed to engage in exercises for laryngeal elevation for a longer period of time than the other group, while patients with HNC needed to engage in vocalization and singing that included syllables and words requiring substantial oral motor movements (e.g., the oral cavity and facial muscles) for more time than the patients with PD.

## 4. Discussion

This study investigated whether a singing-enhanced swallowing protocol led to improvement in swallowing function, speech-language function, and quality of life for patients with dysphagia due to PD or HNC. This study also compared the benefits of the protocol between the two diagnostic groups.

In terms of changes in swallowing function measured by VDS, both patient groups tended to show improvements in swallowing thick solid barium, while improvements in swallowing the liquid type of barium were only observed in the HNC group. Given that voluntary swallowing exercises are important [27], behavioral techniques, including supraglottic swallow, Mendelsohn maneuver, effortful swallow, Masako maneuver, and Shaker exercise, have traditionally involved repeated exercises that strengthen the relevant muscles [28]. These techniques require complex coordination and maintenance of high-intensity exercises [29]. As such, the need for more diversified strategies is increasing, and the patient population itself is becoming more diverse. The results of this study indicate that singing-enhanced protocols are effective in promoting motor coordination (e.g., laryngeal elevation) in patients with dysphagia. Singing, as a complex process involving breathing and oral motor movements, can involve diverse types and levels of coordination of different muscles engaged in the swallowing process with various combinations of breathing patterns and target syllables requiring different oral motor movements [30]. In addition to patients with neurological impairment, this study supports the wider applicability of the singing-enhanced swallowing protocol for patients with structural damage, such as associated with HNC.

It is interesting to note that while both patient groups showed an improved ability in swallowing thick and thin solid barium, an improved ability in swallowing the liquid type of barium was only found in patients with HNC. Such group differences are attributed to the fact that direct damage to the oral motor structure causes swallowing difficulties in patients with HNC, while dysfunction of motor control at the neural level is involved in patients with PD. Although this finding calls for verification through a long-term application of the protocol, the results of this study indicate that high-intensity strengthening of the involved muscles should be the primary intervention focus for patients with dysphagia due to HNC, while range of motor control should be the main focus for dysphagic patients with PD.

In this study, vocal and speech-language functions were measured along with the swallowing function. Given that singing tasks are based on breathing, phonation, and articulation, which are also the main issues for patients with dysphagia, changes in such measures would support how a singing-enhanced protocol can influence patients with dysphagia in multiple domains. Notably, the study results show that laryngeal AMR and L-DDK increased after the intervention compared to before the intervention for all of the patients. These changes align with previous studies applying singing-based interventions for patients with dysphagia due to neurological impairment [12]. The singing tasks in this study targeted laryngeal elevation and were based on articulation of target syllables with demand for more precise coordination, which led to improvements in motor speech control [12,15]. Improvement in the speech-language measures was evidenced in the HNC group, who experienced resection of speech-related structures (e.g., jawbone or tongue), which affected not only their lower-level laryngeal coordination but also their coordination during the oral phase. Although singing-based training to maintain laryngeal elevation was initially developed for neurological patients, this study supports its effective use in more diverse patient populations, including those needing laryngeal muscle rehabilitation due to PD.

In terms of MPT as an index for breathing control, increases were found in patients with PD but not in patients with HNC. This can be explained by differences in respiratory symptoms between the two groups. Patients with PD show problems in the breathing control required for speech production [31,32], which is related not only to speech disorders but also swallowing disorders. Patients with HNC show relatively less impairment in their respiratory control. Accordingly, engagement in singing demands more of patients with PD than HNC. The slight decrease in MPT observed in patients with HNC may be partially attributed to the ceiling effect because these patients exhibited MPTs in the normal range prior to the study. Furthermore, structured singing tasks with an emphasis on respiratory control may effectively address muscle coordination, which leads to greater benefits for patients with PD whose symptoms are directly related to such control issues. Controlling their singing within music-based timing structures might require intentional coordination of breathing patterns for patients with PD, while a higher demand may be involved in exercising oral motor movements and facial muscles related to articulation for patients with HNC.

All of the patients showed increased SWAL-QOL scores. This is indicative of the functional effects of the singing intervention and its psycho-emotional impact. Reports from the patients also indicated positive changes in their perception of their voice. They reported that they had a negative perception of their voice before the intervention and had little experience with musical performance, but they were able to increasingly experience positive benefits from singing as the intervention progressed. Specifically, they reported improved confidence in their singing and vocal functions as a result of the musical intervention. These results support the psycho-emotional benefits of a singing-based intervention for patients with dysphagia, including positive changes in their perception of their own voice, which may have clinical implications, particularly for patients with structural damage.

Finally, this study analyzed the repetition rate of each component within the protocol and compared the rate depending on the diagnosis of the patients (i.e., PD versus HNC). Given that a higher repetition rate of a particular component indicates lower performance and greater demand for that component, a comparison of such rates between the PD patients and those with HNC highlights which components required more effort by the different patient groups. Based on this information, the intensity level of a specific component in the protocol could be adjusted for targeting a particular patient population, more specifically the cause of the dysphagia. In this study, while patients with PD required more repetition of respiratory exercises, laryngeal elevation, and modified singing tasks targeting respiratory control, patients with HNC needed to spend more time completing the modified singing tasks related to articulatory control at the expected level. This highlights how singing-based interventions may need to be differentially implemented for different patient groups, even though the swallowing difficulties they experience are similar. This is also supported by the fact that dysphagia due to PD is more related to involuntary abnormal movements during swallowing and related processes [33] and that dysphagia due to HNC is more related to deterioration of oral and pharyngeal muscles, particularly due to treatment (e.g., radiation therapy) [34]. This study suggests that the cause of a patient’s dysphagia should be considered when determining the components of a singing-based intervention. Such a consideration makes it possible to provide more targeted interventions within a limited period of time that are more intensive and efficient for the patient.

Despite the promising results of this study, readers are cautioned against generalizing the findings due to the study’s small sample size. While this study presents clinical implications for what should be considered in the design of a singing-enhanced swallowing protocol, the efficacy of such a protocol must be demonstrated through future studies with larger sample sizes and more diverse patient populations.

## 5. Conclusions

This study investigated the applicability of a singing-enhanced swallowing protocol for diverse populations (i.e., patients with dysphagia due to HNC). It also examined the differential benefits for two different diagnostic groups (i.e., patients with dysphagia due to neurological damage versus patients with dysphagia due to structural damage). Intervention activities focused on relaxation of respiratory muscles and auxiliary respiratory muscles through physical activities, vocalization preparation, and vocalization and modified singing to target respiratory control, oral motor control, and laryngeal elevation in an integrated manner.

Since singing can be used for a wide range of therapeutic purposes, more detailed specification of which singing activities best support specific problems and diagnoses is needed. Furthermore, it is important to note that the objective of singing and the contents of the intervention’s activities were selected based on the underlying cause of dysphagia and the specific problems each patient was experiencing. This highlights the need for further specification and systematization of therapeutic singing activities for treating the numerous symptoms of patients with dysphagia based on their etiology. It is hoped that future research will propose more efficient compositions for music interventions and more specific application methods for treating patients with dysphagia arising from diverse causes.

## Figures and Tables

**Table 1:** Demographic information of participants.

Patient	Sex	Age	Diagnosed Disorder	Duration Since Diagnosis (Years)
A	Female	77	Parkinson’s disease	5.2
B	Male	72	Multiple system atrophy	7.0
C	Female	54	Tongue cancer	3.0
D	Female	60	Mandibular gland cancer	4.8

**Table 2:** Results of VDS measurements.

Food Texture	Volume	Swallowing Phase	Dysphagia Patients with PD						Dysphagia Patients with HNC					
			A			B			C			D		
			Pre	Post	Change	Pre	Post	Change	Pre	Post	Change	Pre	Post	Change
Thick solid	5 cc	Oral	28.0	26.5	+	21.0	19.0	+	18.0	15.0	+	18.5	13.0	+
		Pharyngeal	43.0	32.0	+	38.5	37.0	+	26.0	21.5	+	32.5	2.0	+
		Total	71.0	58.5	+	59.5	56.0	+	44.0	36.5	+	51.0	15.0	+
Thick solid	15 cc	Oral	24.0	19.5	+	16.5	16.5	/	19.5	18.0	+	14.0	10.0	+
		Pharyngeal	43.0	41.0	+	43.0	40.0	+	26.0	23.5	+	22.0	2.0	+
		Total	67.0	60.5	+	59.5	56.5	+	45.5	41.5	+	36.0	12.0	+
Thin solid	5 cc	Oral	28.0	23.5	+	16.5	13.5	+	15.0	15.0	/	15.5	10.0	+
		Pharyngeal	40.0	40.0	/	38.5	35.5	+	30.5	12.5	+	26.5	2.0	+
		Total	68.0	63.5	+	55.0	49.0	+	45.5	27.5	+	42.0	12.0	+
Thin solid	15 cc	Oral	25.0	22.0	+	12.0	12.0	/	15.0	15.0	/	14.0	8.5	+
		Pharyngeal	35.5	32.5	+	34.0	34.0	/	30.5	14.5	+	26.5	11.0	+
		Total	60.5	54.5	+	46.0	46.0	/	45.5	29.5	+	40.5	19.5	+
Liquid	5 cc	Oral	18.0	18.0	/	15.0	15.0	/	22.0	15.5	+	10.0	8.5	+
		Pharyngeal	30.5	30.5	/	30.5	30.5	/	30.5	6.5	+	2.0	2.0	/
		Total	48.5	48.5	/	45.5	45.5	/	52.5	22.0	+	12.0	10.5	+
Liquid	15 cc	Oral	16.5	16.5	/	13.5	13.5	/	22.0	15.5	+	8.5	8.5	/
		Pharyngeal	30.5	30.5	/	30.5	30.5	/	21.5	21.5	/	2.0	2.0	/
		Total	46.5	46.5	/	43.5	43.5	/	43.5	37.0	/	10.5	10.5	/

Note. Data in the table are the scores of VDS. Shaded area and + represent improved swallowing function, given that the increased VDS score indicates increased severity of dysphagia, / indicates no change at posttest compared to pretest.

**Table 3:** Results of vocal and speech-language function measurements.

Measurement	Patients with PD						Patient with HNC					
	A			B			C			D		
	Pre	Post	Change	Pre	Post	Change	Pre	Post	Change	Pre	Post	Change
MPT (seconds)	13.3	16.0	+	3.7	8.8	+	10.5	9.8	−	14.2	12.0	−
AMR (times)												
/puh/	8	9	+	6	7	+	14	18	+	19	24	+
/tuh/	7	9	+	6	7	+	17	17	/	19	24	+
/kuh/	8	12	+	5	6	+	15	16	+	18	24	+
SMR (times)	4	6	+	2	2	/	7	7	/	6	8	+
L-DDK (times)	10	13	+	5	8	+	14	20	+	15	20	+

Note. Shaded area and + indicate improvement; − indicates decreases in measures and / indicates no change from pretest to posttest.

**Table 4:** Evaluation scores for SWAL-QOL.

Patient	A		B		C		D	
Measurement	Pre	Post	Pre	Post	Pre	Post	Pre	Post
Burden	2	4	4	8	4	5	2	4
Eating duration	2	4	6	7	3	6	3	6
Eating desire	11	10	12	11	10	9	9	10
Symptom frequency	33	37	43	46	27	40	25	31
Food selection	2	5	8	7	5	5	6	8
Communication	4	4	6	8	5	5	4	7
Fear	5	8	14	15	9	12	6	10
Mental health	5	10	20	19	8	11	7	9
Social functioning	7	10	20	25	10	10	10	13
Fatigue	3	3	6	10	8	9	6	5
Sleep	5	5	4	7	6	6	4	6
Total	79	100	143	163	95	118	82	109
